# A suicide in a Russian novel

**DOI:** 10.1177/10398562241307801

**Published:** 2024-12-17

**Authors:** Saxby Pridmore

**Affiliations:** Hobart, TAS

Dear Editor,

Fiction gives insights into the culture of the time at which it was written. Ivan Turgenev was a successful novelist, born in Russia in 1818. His last novel, ‘Virgin Soil’ was completed in 1877. It is set in the 1870s – during the ‘Populist movement’ (1860s and 70s) which promoted ‘the people’ rising up against ‘the elite’. The culmination is the suicide of the main character Nejdanov (Nezhdanov), the illegitimate son of an aristocrat.

Nejdanov, a young adult student, a convert to the movement, takes a position as a child’s tutor in a country area. His employer and those he meets are also converts. He meets Mariana a strong believer, and they fall in love and talk of marriage. They and some others have conversations with those they meet locally and encourage ‘the people’ to rise up in rebellion. Suddenly, the authorities commence imprisoning the ‘revolutionaries’ they can identify.

Nejdanov and Mariana meet, and it is expected they will escape the region. However, Nejdanov states he no longer believes in ‘the cause’. Knowing Mariana is deeply committed to the cause, and himself (and will not leave him) to solve this predicament, he announces the intention and kills himself by pistol shot.

‘Virgin Soil’ tells that in 1877 in Russia, suicide could be triggered by complex predicaments. In this case, one member of a romantically connected couple lost conviction to a cause which they had once shared. He died by suicide so that his partner could move on in life and he could escape the vengeance of the authorities and his own feeling of inadequacy and guilt. Such circumstance underpins much suicide in the present era.

